# Supplementary material for: Mapping the evolution of fertility support policies in China: A content and instrumental analysis
Source: PLoS One. 2025 Oct 9;20(10):e0332137. doi: 10.1371/journal.pone.0332137 (PMC12510515; doi:10.1371/journal.pone.0332137)
Supplement: S1 Appendix — (ZIP) [file pone.0332137.s001.zip › S1 Appendix. 226 original policy documents/23-国家卫生计生委办公厅关于印发中共中央国务院关于实施全面两孩政策改革完善计划生育服务管理的决定学习宣传提纲的通知(FBM-CLI-4-263178).doc]

国家卫生计生委办公厅关于印发中共中央国务院关于实施全面两孩政策改革完善计划生育服务管理的决定学习宣传提纲的通知

**发布部门：** [国家卫生和计划生育委员会(已撤销)](https://www.pkulaw.com/law/chl?Aggs.IssueDepartment=60319) [机构沿革](javascript:void(0))

**发文字号：**国卫办指导函〔2016〕38号

**发布日期：**2016.01.15

**实施日期：**2016.01.15

**时效性：** [现行有效](https://www.pkulaw.com/law/chl?Aggs.TimelinessDic=01)

**效力级别：** [部门规范性文件](https://www.pkulaw.com/law/chl?Aggs.EffectivenessDic=XE0302)

**法规类别：** [计划生育管理](https://www.pkulaw.com/law/chl?Aggs.Category=09705)

国家卫生计生委办公厅关于印发中共中央 国务院关于实施全面两孩政策改革完善计划生育服务管理的决定学习宣传提纲的通知
（国卫办指导函〔2016〕38号）

各省、自治区、直辖市卫生计生委，新疆生产建设兵团卫生局、人口计生委，解放军、武警部队计划生育领导小组办公室，中直机关、中央国家机关人口计生委，委机关各司局，委直属和联系单位：
　　现将《[中共中央 国务院关于实施全面两孩政策改革完善计划生育服务管理的决定](https://www.pkulaw.com/chl/8564756bae805261bdfb.html)》学习宣传提纲印发给你们，供参考。

国家卫生计生委办公厅
2016年1月15日

　　《中共中央 国务院关于实施全面两孩政策 改革完善计划生育服务管理的决定》学习宣传提纲

　　**一、**《决定》发布的重大意义
　　党中央、国务院历来重视人口和计划生育工作。1980年，党中央发表《关于控制我国人口增长问题致全体共产党员、共青团员的公开信》。为加强人口和计划生育工作，党中央、[国务院先后于1991年作出《关于加强计划生育工作严格控制人口增长的决定](https://www.pkulaw.com/chl/8d47b50a52dd0cafbdfb.html)》，2000年作出《[关于加强人口与计划生育工作稳定低生育水平的决定](https://www.pkulaw.com/chl/f827fcaffffe8d79bdfb.html)》，2006年作出《[关于全面加强人口和计划生育工作统筹解决人口问题的决定](https://www.pkulaw.com/chl/5e7b01010a4102ffbdfb.html)》，明确了各个阶段的工作思路、目标和任务，形成了齐抓共管的良好局面，有力推动了人口和计划生育事业的持续健康发展。
　　以习近平同志为总书记的党中央高度重视人口与经济社会的协调和可持续发展，党的十八届三中全会作出了“坚持计划生育的基本国策，启动实施一方是独生子女的夫妇可生育两个孩子的政策，逐步调整完善生育政策，促进人口长期均衡发展”的决策部署，党中央、国务院印发了《关于调整完善生育政策的意见》。党的十八届五中全会作出了“促进人口均衡发展。坚持计划生育的基本国策，完善人口发展战略。全面实施一对夫妇可生育两个孩子政策”的总体部署，这是对人口和计划生育政策的一次重大调整，具有里程碑意义，为新时期人口和计划生育改革与发展指明了方向。
　　《[中共中央 国务院关于实施全面两孩政策改革完善计划生育服务管理的决定](https://www.pkulaw.com/chl/8564756bae805261bdfb.html)》（以下简称[《决定》](https://www.pkulaw.com/chl/8564756bae805261bdfb.html)），对过去40多年计划生育工作成就给予了充分肯定，深刻分析了我国人口发展的新形势，在新的历史起点上，部署了生育政策调整工作，明确提出了计划生育改革发展的思路、目标、原则、主要任务和措施，是贯彻落实五中全会精神、指导“十三五”和今后一个时期我国人口和计划生育工作的纲领性文件。
　　认真学习贯彻落实[《决定》](https://www.pkulaw.com/chl/8564756bae805261bdfb.html)精神，不断探索新形势下落实计划生育基本国策的体制机制和方式方法，使计划生育成为惠及亿万家庭的甜蜜事业，对于全面建成小康社会、实现中华民族伟大复兴的中国梦具有十分重要的意义。

　　**二、**[《决定》](https://www.pkulaw.com/chl/8564756bae805261bdfb.html)的框架和重点内容
　　[《决定》](https://www.pkulaw.com/chl/8564756bae805261bdfb.html)分为六个部分：
　　一是重大意义。充分肯定了40多年来计划生育工作取得的巨大成就和广大人民群众、计划生育家庭、计划生育工作者作出的重要贡献，分析了当前我国人口形势呈现的重大转折性变化，阐明了实施全面两孩政策、改革完善计划生育服务管理的重要性。
　　二是指导思想、原则和目标。提出了以增进家庭和谐幸福、促进人口长期均衡发展为主线，坚持计划生育基本国策，实施全面两孩政策、推进计划生育服务管理改革的总体思路，强调了“以人为本、创新发展、法治引领、统筹推进”的原则，明确了计划生育改革发展的主要目标。
　　三是稳妥扎实有序实施全面两孩政策。依法修订地方条例及相关法规，组织实施一对夫妇可生育两个孩子政策。改革生育服务管理制度，加强出生人口监测预测，合理配置公共服务资源，满足新增公共服务需求。
　　四是大力提升计划生育服务管理水平。加强妇幼健康计划生育服务，推进流动人口基本公共卫生计生服务均等化，强化基层基础工作，充分发挥社会组织作用。
　　五是构建有利于计划生育的家庭发展支持体系。加大对计划生育家庭扶助力度，增强家庭抚幼和养老功能，促进社会性别平等。
　　六是切实加强组织领导。坚持党政一把手亲自抓、负总责，坚持计划生育目标管理责任制，坚持“一票否决”制，加强部门协作，深化人口发展战略研究，做好宣传和舆论引导，抓好督导落实。

　　**三、**[《决定》](https://www.pkulaw.com/chl/8564756bae805261bdfb.html)的主要精神
　　[《决定》](https://www.pkulaw.com/chl/8564756bae805261bdfb.html)明确了计划生育改革发展的主线。坚持计划生育基本国策、增进家庭和谐幸福、促进人口长期均衡发展，这一主线和精神始终贯穿[《决定》](https://www.pkulaw.com/chl/8564756bae805261bdfb.html)全文。坚持计划生育基本国策是前提和基础，增进家庭和谐幸福是调整政策、推进改革的出发点和落脚点，促进人口长期均衡发展是改革发展的方向和目标。
　　[《决定》](https://www.pkulaw.com/chl/8564756bae805261bdfb.html)突出了实施全面两孩政策和改革完善计划生育服务管理两大任务。实施全面两孩政策是人口和计划生育政策的重大调整，落实好党中央的决策，必须配套推进计划生育服务管理改革，改进生育服务管理，提升妇幼健康计划生育服务能力，健全家庭发展支持政策，创新计划生育体制机制。
　　[《决定》](https://www.pkulaw.com/chl/8564756bae805261bdfb.html)确立了新时期计划生育工作的原则。坚持以人为本，尊重家庭在计划生育中的主体地位，引导群众负责任、有计划地生育；坚持法治引领，充分发挥立法对完善生育政策和服务管理改革的引领、规范和保障作用；坚持统筹推进，实现调整完善生育政策与服务管理改革同步推进、配套政策措施同步制定；坚持创新发展，促进人口和计划生育工作的“三个转变”，即由控制人口数量为主向调控总量、提升素质和优化结构并举转变，由管理为主向更加注重服务家庭转变，由主要依靠政府力量向政府、社会和公民多元共治转变。
　　[《决定》](https://www.pkulaw.com/chl/8564756bae805261bdfb.html)提出了新时期人口和计划生育改革发展目标。总目标是努力实现规模适度、素质较高、结构优化和分布合理的人口均衡发展，促进人口与经济社会、资源环境协调可持续发展，为实现中华民族伟大复兴的中国梦提供坚实基础和持久动力。阶段性目标是：到2020年，计划生育服务管理制度和家庭发展支持体系较为完善，政府依法履行职责、社会广泛参与、群众诚信自律的多元共治格局基本形成，计划生育治理能力全面提高；覆盖城乡、布局合理、功能完备、便捷高效的妇幼保健计划生育服务体系更加完善，基本实现人人享有计划生育优质服务，推动联合国2030年可持续发展议程的落实；保持适度生育水平，人口总量控制在规划目标之内。

　　**四、**计划生育取得了举世瞩目的伟大成就
　　坚持计划生育基本国策，体现了几代中央领导集体的远见卓识，是符合我国基本国情的重大决策。计划生育是强国富民安天下的伟大事业，功在当代，利在千秋，成就来之不易，应当倍加珍惜。
　　客观认识我国推行计划生育的历史背景。新中国成立后，我国经济恢复，社会安定，人民生活改善，人口死亡率尤其是婴儿死亡率大幅下降，人均预期寿命延长，人口快速增长。我国大陆总人口从5.4亿迅速增加到1970年的8.3亿，1980年的9.9亿，1981年达10.01亿，年均自然增长率高达20‰，广大人民群众在衣食住行、就学就医就业等方面的困难日益突出。上世纪70年代，为应对人口过快增长给国家发展、人民生活、资源环境带来的巨大压力，满足城乡群众对节育的现实需求，我国开始在城乡全面推行计划生育。1980年，党中央发表《关于控制我国人口增长问题致全体共产党员、共青团员的公开信》，1982年，计划生育被确立为基本国策。
　　充分肯定计划生育对经济社会发展的巨大作用。计划生育在国家富强、社会进步、民族振兴的过程中发挥了不可估量的作用。一是有效控制了人口过快增长的势头，实现了人口再生产类型的历史性转变。人口自然增长率从1971年的23.3‰下降到2014年的5.21‰，总和生育率由5.4下降到1.5-1.6。二是有效缓解了人口对资源环境的压力，改善了人民群众的生存和发展状况。如果不实行计划生育，人均耕地、粮食、森林、淡水资源等会比目前低20%以上，人民群众生活质量不可能达到今天的水平。2014年孕产妇死亡率降至21.7/10万，婴儿死亡率降至8.9‰。三是创造了有利于经济增长的“人口红利期”，为改革开放、经济快速发展作出了巨大贡献。四是为世界人口发展和减贫事业作出了重大贡献，树立了负责任大国的良好形象。
　　充分肯定广大计划生育干部付出的艰辛努力和亿万计划生育家庭作出的巨大贡献。计划生育是一场破旧立新、移风易俗的社会革命，是“天下第一难事”。40多年来，广大计生干部在各级党委政府领导下，广泛宣传党和国家的计划生育政策，深入细致开展群众思想工作，充分发动群众、依靠群众自觉落实计划生育。在这个过程中，广大计生干部特别是基层计生工作者，走遍千山万水，访遍千家万户，说尽千言万语，付出千辛万苦，为贯彻基本国策付出了大量心血和汗水。广大党员、干部、共青团员用实际行动响应党中央的号召，带头实行计划生育；亿万人民群众顾全国家大局，克服实际困难，自觉实行计划生育，为国家发展作出了巨大贡献。

　　**五、**坚持计划生育基本国策
　　人口问题是人类社会共同面对的基础性、全局性和战略性问题。坚持计划生育基本国策是我国立足国情、实现可持续发展的战略选择。
　　充分认识坚持计划生育基本国策的重要性、长期性。人口众多、资源相对不足、环境容量有限、城乡区域发展不平衡是我国的基本国情。2014年末我国总人口为13.68亿人，尽管生育水平和人口自然增长率已经下降到较低水平，但未来十几年总人口还将继续增长。预计2030年前后总人口达到14.5亿左右的峰值。我国人居环境适宜地区仅占国土面积的45%左右，人均耕地、淡水、石油储量分别为世界平均水平的40%、24%、10%。随着工业化、城镇化快速推进，经济总量不断扩大，能源资源需求还将持续增长。到本世纪中叶，我国人口总量仍将保持在13.8亿人左右，人口众多的基本国情不会根本改变，人口对经济社会发展的压力不会根本改变，人口与资源环境的紧张关系不会根本改变，必须长期坚持计划生育基本国策，保持适度的人口规模，促进人口与经济社会、资源环境的协调和可持续发展。
　　新形势新使命赋予计划生育基本国策新的内涵。我国人口发展的内在动力和外部条件已经发生了显著变化。人口总量增长势头明显减弱，劳动年龄人口和育龄妇女开始减少，老龄化程度不断加深；群众生育观念发生重大转变，少生优生已成为社会生育观念的主流；家庭规模趋向小型化，养老抚幼功能弱化；人口红利减弱，以人力资本为核心的国际竞争优势有待进一步加强。这些变化对人口安全和经济社会发展带来新的挑战，必须从全局和战略高度出发，立足国情，遵循规律，正确处理当前与长远、总量与结构、人口与资源环境的关系，逐步调整完善生育政策，促进人口长期均衡发展。实施全面两孩政策，改革完善计划生育服务管理，不是对过去工作的否定，而是对计划生育的坚持和完善。当前，坚持计划生育基本国策，就是要实施好全面两孩政策，推进生育服务管理改革，引导家庭负责任、有计划地安排生育；做好避孕节育、优生优育、生殖健康、妇幼保健各项服务，提高出生人口素质和母婴健康水平；完善家庭发展政策，鼓励按政策生育，保障计划生育家庭的权益，加大困难家庭帮扶力度；调控人口总量，提升人口素质，优化人口结构，引导人口合理分布，促进家庭和谐幸福和人口均衡发展。

　　**六、**稳妥扎实有序实施全面两孩政策
　　实施全面两孩政策关系全局、关系长远、关系千家万户，是站在中华民族长远发展的战略高度促进人口均衡发展的重大举措。
　　充分认识实施全面两孩政策的重大意义。一是有利于更好地满足群众生育意愿，促进家庭幸福与社会和谐。据调查，20～44岁已婚人群平均理想子女数为1.93个。实施全面两孩政策，能够满足绝大多数群众的生育意愿。从长期看，政策调整后，家庭规模将有所扩大，有利于改善家庭结构，增强家庭代际支持、养老照料功能，促进家庭发展。二是有利于优化人口结构，减缓老龄化压力，增加劳动力供给。据预测，实施全面两孩政策，2050年可增加约3000万劳动力，劳动力年龄结构有所改善；到2050年老年人口在总人口中所占比重，与不调整政策相比降低2个百分点，将在一定程度上减缓人口老龄化进程。三是有利于促进经济持续健康发展。新增人口进入劳动年龄后，将降低人口抚养比，提高潜在经济增长率，稳定经济增长预期。
　　实施全面两孩政策时机成熟、条件具备。一是从劳动年龄人口变动看，今后三四十年内，我国劳动力总量都将比较充裕。我国15-64岁劳动年龄人口2013年达到峰值10.06亿，2015年为10亿。预计2020年、2030年分别为9.9亿、9.58亿，到2050年还有8.27亿。二是从社会抚养负担变动看，未来一段时期我国仍处于有利于经济发展的人口红利期。2014年，我国人口总抚养比为36.2%，“十三五”时期虽有所升高，但幅度不大，预计到2030年抚养比仍低于50%。三是从育龄妇女变动看，有利于平缓出生人口波动。2011年我国育龄妇女数量达到峰值3.8亿，预计2020年减少到3.3亿，其中20-29岁生育旺盛期育龄妇女数量从2012年的峰值1.15亿减少到2020年的8000万。此外，单独两孩政策的实施释放了部分生育势能，有助于准确了解群众生育意愿和生育行为，为实施全面两孩政策积累了经验。
　　依法组织实施全面两孩政策，确保政策平稳落地。根据修改后的《[人口与计划生育法](https://www.pkulaw.com/chl/18c6c388fc14eaa9bdfb.html)》，全面两孩政策自2016年1月1日起在全国各地同步实施。各省（区、市）人口与计划生育条例和相关规定要作相应修改。各省（区、市）人民政府综合评估本地人口发展形势、计划生育工作基础和政策实施风险，科学制定实施方案并报国务院主管部门备案。各地要确保政策平稳落地，生育水平不出现大幅波动。同时要根据生育服务需求和人口变动情况，合理配置妇幼保健、儿童照料、学前和中小学教育、社会保障等资源，引导和鼓励社会力量举办非营利性妇女儿童医院、托儿所、幼儿园等服务机构，满足新增公共服务需求。

　　**七、**改革生育服务管理制度
　　党的十八届三中全会以来，卫生计生系统以启动实施单独两孩政策为契机，以生育服务证改革为抓手，改进服务管理，方便群众办事，产生了良好的社会反响。实施全面两孩政策，必须继续深化生育服务管理制度改革。
　　一是实行生育登记服务制度。对生育两个及两个以内孩子的，不实行审批，由家庭自主安排生育。优化再婚等特殊情形再生育办理流程，推行网上办事、一站式服务和承诺制，为群众提供高效便捷服务。
　　二是加强出生人口监测预测。完善信息动态采集机制，加强人口变动情况调查，建立出生人口监测和预警机制。加快推进国家人口基础信息库和人口健康信息化建设，实现跨区域信息互联互通和跨部门信息共享。
　　三是依法依规处理政策外生育行为。对政策外多孩生育的，依法征收社会抚养费，是国家工作人员的，依法给予行政处分，其他人员还应当由其所在单位或者组织给予纪律处分。对政策实施前已征收社会抚养费的，维持原处理决定；对尚未处理或已作出处理决定但尚未征收到位的，依法依规妥善处理。加强和规范社会抚养费征收管理，严格落实“收支两条线”。

　　**八、**加强妇幼健康计划生育服务
　　健全妇幼保健计划生育服务体系是落实计划生育基本国策的重要保障。要把提高妇幼健康服务能力、服务计划生育家庭、实现人人享有生殖健康服务作为工作重心，不断提升服务质量，提高人民群众的满意度和获得感。
　　一是推进优生优育全程服务。为妇女儿童提供系统、规范的孕前保健、孕期保健、住院分娩、儿童保健和计划免疫等服务。推进出生缺陷综合防治，提高出生人口素质。落实免费孕前优生健康检查，开展产前筛查和产前诊断服务，提高全国新生儿疾病筛查率。向不孕不育等生育困难人员提供必要的人类辅助生殖技术服务。做好孕产妇系统管理，确保母婴安全。
　　二是提供计划生育优质服务。为实行计划生育的育龄夫妻提供免费基本计划生育技术服务。加强再生育咨询指导，为计划生育人群提供取环、复通等服务。推进避孕节育知情选择，向育龄人群提供安全、有效、适宜的安全避孕服务，提高服务的公平性和可及性。加强基础研究和科技创新，开发推广避孕节育、优生优育、妇幼保健和生殖健康的新技术新产品，促进成果转化。
　　三是大力推进服务体系建设。推进妇幼保健计划生育服务机构标准化建设和规范化管理。加强基层医疗卫生机构和公立医院妇幼健康服务能力建设。鼓励和引导社会资本举办妇女儿童医院，满足人民群众日益增长的多层次、多样化医疗服务需求。加强孕产妇和新生儿危急重症救治能力建设，畅通危急重症救治绿色通道，建成分级负责、上下联动、应对有序、运转高效的孕产妇和新生儿危急重症急救、会诊、转诊网络。
　　四是加强产儿科紧缺专业人才培养和使用。在有条件的高校探索开设儿科专业，开展大学本科助产相关专业招生培养工作。在住院医师规范化培训中加大儿科等紧缺专业招收规模。开展助产士、儿科医师转岗培训，鼓励医护人员向助产士、儿科医师专业方向发展。结合公立医院医疗服务价格改革，合理调整体现医务人员技术劳务价值的医疗保健服务价格。建立助产士、产科医师、儿科医师等紧缺人才激励机制，在薪酬分配方面给予倾斜，改善医务人员待遇，增加岗位吸引力。

　　**九、**推进流动人口基本公共卫生计生服务均等化
　　未来十几年，我国仍处于城镇化快速发展阶段，人口流动迁移保持活跃。流动人口在现居住地婚恋、生育、养育子女的比例大幅增加，计划生育、优生优育、生殖健康服务需求不断增长。推进流动人口基本公共卫生计生服务均等 化，是确保人人享有妇幼保健和计划生育服务的重要任务。
　　一是按照常住人口配置服务资源，将流动人口纳入城镇基本公共卫生和计划生育服务范围。全面落实国家规定的免费卫生计生服务项目。加强职业健康、传染病防治、心理健康、避孕节育、妇幼保健、生殖健康服务。
　　二是巩固完善流动人口信息互通、服务互补、管理互动的全国“一盘棋”工作机制。推进网上信息核查和共享，做好流动人口在居住地的生育登记服务。修订完善流动人口服务管理法规。加强流动人口统计监测。
　　三是广泛开展生殖健康科普宣传，增强流动人口自我保健意识和防护能力。实施流动人口健康促进行动计划，提高流动人口健康素养和健康水平。以农民工、青少年、妇女、儿童为重点人群，开展有针对性的健康教育和干预项目。
　　四是关怀关爱流动人口和留守人群，促进社会融合。开展流动人口社会融合示范试点工作，提高流动人口家庭发展能力。建立和完善留守妇女、儿童、老人关爱服务体系，实施留守儿童健康促进等项目。

　　**十、**强化基层基础工作
　　计划生育工作的重心在基层。要完善宣传倡导、依法管理、优质服务、政策推动、综合治理的计划生育长效工作机制。把稳定和健全基层计划生育行政管理、技术服务和群众工作网络作为改革的着力点，有效整合资源，夯实基层基础，最大限度地调动基层的积极性、创造性。
　　一是稳定行政管理机构和队伍。实施全面两孩政策后，计划生育工作要加强政策和科普知识宣传，为群众提供便捷优质的婚育健康全程服务，落实计划生育奖励扶助政策，加大对存在困难的计划生育家庭的帮扶力度，做好政策衔接和出生人口监测，依法依规查处政策外多孩生育。计划生育服务管理呈现出许多新的特点，任务更重、要求更高，必须稳定和加强基层计划生育工作力量。要增强卫生计生综合监督行政执法能力，健全乡镇（街道）计生办，按照常住人口比例配置工作人员。
　　二是优化配置县乡服务机构和队伍。有效整合县、乡妇幼保健和计划生育技术服务资源，保障计划生育宣传教育、技术服务、优生指导、药具发放、信息咨询、随访服务、生殖保健、人员培训等职责的落实。
　　三是加强村（居、社区）队伍建设。健全村（居）计划生育领导小组（计划生育委员会）、计划生育协会，明确村（居）党支部、村（居）委会的计划生育职责。妥善解决村级计生专干的报酬待遇、养老保障和其他历史遗留问题。

　　**十一、**充分发挥社会组织作用
　　政府与社会协同治理是新形势下落实计划生育基本国策的有效途径。要按照简政放权、放管结合、优化服务的要求，切实转变政府职能，充分发挥计生协等群团和其他社会组织作用，提高计划生育综合治理能力。
　　一是加强政府与社会协同治理。强化政府制定政策、规划和监管的职责，逐步将适合的计划生育服务管理事项交由社会组织承担。加大政府购买服务力度，在计划生育宣传咨询、青少年和流动人口性与生殖健康教育、避孕药具发放等方面，逐步扩大购买服务范围。广泛动员工会、共青团、妇联、中国计生协等群团组织以及中国人口福利基金会等社会组织共同做好计划生育工作。鼓励社会组织依法开展人口和计划生育公益慈善和帮扶救助活动。
　　二是充分发挥计划生育协会的生力军作用。切实加强县、乡级计划生育协会的组织和能力建设，提升专业化、职业化水平。通过项目运作、购买服务等方式，更多地把直接面向群众的宣传教育、生殖健康咨询服务、优生优育指导、计划生育家庭帮扶、权益维护和流动人口服务等工作交由协会承担。在城乡社区和企事业单位，引导群众广泛开展计划生育自我管理、自我服务、自我教育、自我监督。

　　**十二、**完善计划生育家庭奖励和扶助政策
　　按照“老人老办法，新人新办法”的原则，做好全面两孩政策实施前后计划生育家庭奖励和扶助政策的衔接，切实保障计划生育家庭的合法权益。
　　一是完善各项计划生育家庭奖励和扶助政策。对政策调整前的独生子女家庭和农村计划生育双女家庭，继续实行已有的各项奖励扶助政策，在社会保障、集体收益分配、就业创业、新农村建设等方面予以倾斜。完善计划生育家庭奖励扶助和特别扶助制度，建立扶助标准动态调整机制。推进计划生育与扶贫开发相结合，继续实施“少生快富”工程。
　　二是关怀帮扶存在特殊困难的计划生育家庭。在国家提倡一对夫妻生育一个子女期间自愿终身只生育一个子女的夫妻，独生子女发生意外伤残、死亡的，按照规定获得扶助。2013年底，国家卫生计生委、民政部、财政部、人力资源社会保障部、住房城乡建设部等5部门联合下文，从经济扶助、养老保障、医疗保障、社会关怀等方面作出了多项制度安排，进一步明确了地方政府的责任。各地要制定具体政策措施，切实落实帮扶责任。
　　三是政策调整后自愿生育一个孩子的家庭不再享受奖励扶助和优待政策。全面两孩政策实施后自愿只生育一个孩子的夫妇，不再发放《独生子女父母光荣证》，不再享受独生子女父母奖励费和各种优先优惠政策。独生子女家庭再生育的，停止享受独生子女父母奖励政策，此前已享受的不退还。政策调整后自愿生育一个孩子的父母，不再纳入奖励扶助和特别扶助范围。

　　**十三、**增强家庭抚幼和养老功能
　　家庭是社会的基本细胞，是实行计划生育的主体。要注重家庭发展，建立完善包括生育支持、幼儿养育、青少年发展、老人赡养、病残照料等在内的家庭发展支持政策。
　　一是完善计划生育奖励假制度。各省（区、市）在修订地方人口与计划生育条例时，按照《[人口与计划生育法](https://www.pkulaw.com/chl/18c6c388fc14eaa9bdfb.html)》，完善计划生育奖励假的规定，制定符合政策生育夫妇延长生育假奖励的相关政策措施，保障其休假期间的合法权益。
　　二是推进医疗卫生与养老服务相结合。建立健全医疗卫生机构与养老机构合作机制，为老年人提供住院治疗、康复护理、生活照料、临终关怀等健康养老服务。推动医疗卫生服务延伸至社区、家庭，为居家老年人提供连续性健康管理服务。鼓励有条件的地方探索建立长期护理保险制度，积极探索多元化的保险筹资模式，保障老年人长期护理服务需求。
　　三是广泛开展创建幸福家庭活动和实施新家庭计划。全面开展创建幸福家庭活动。实施“新家庭计划”项目和计划生育家庭科学育儿、青少年健康发展和养老照护试点，探索健全家庭健康服务体系，增强社区幼儿照料、托老日间照料和居家养老等服务。加大对残疾人家庭、贫困家庭和独居老人的帮扶支持力度。

　　**十四、**促进社会性别平等
　　男女平等是我国的基本国策，也是人口均衡发展的内在要求。自20世纪80年代以来，我国出生人口性别比持续偏高。治理出生人口性别比偏高问题，需要标本兼治，促进社会性别平等。
　　一是深入开展关爱女孩行动，营造有利于女孩成长成才的社会环境。把促进社会性别平等和扶贫帮困相结合，引导群众树立性别平等、生男生女一样好的婚育观。加强对女童教育、女性就业、女孩家庭养老等问题的帮扶，提高女孩家庭的发展能力。
　　二是综合治理出生人口性别比偏高问题。定期开展专项整治行动，依法严厉打击非医学需要的胎儿性别鉴定和选择性别人工终止妊娠行为。提高出生人口性别统计监测水平。
　　三是加大妇女权益保障力度。保障女性宅基地、房屋等财产继承权和土地承包权。加强劳动执法监督，保障女性就业、休假等合法权益，落实生育保险相关待遇。支持女性生育后重返工作岗位，鼓励用人单位制定有利于职工平衡工作与家庭关系的措施。

　　**十五、**切实加强组织领导
　　我国在长期的计划生育实践中，形成了较为健全的计划生育工作领导机制，有力地保障了各项任务的落实。实施全面两孩政策、改革完善计划生育服务管理涉及观念转变、政策衔接和制度创新，必须进一步加强组织领导。
　　一是坚持一把手亲自抓、负总责，落实党政责任。各级党委政府要把计划生育摆在重要位置，将实施全面两孩政策、改革完善计划生育服务管理作为全面深化改革的重要任务，加强统筹协调、政策完善和工作落实。坚持和完善计划生育目标管理责任制，确保责任到位、措施到位、投入到位。对人口和计划生育工作主要目标任务未完成、严重弄虚作假、违法行政造成恶劣影响等情形，实行“一票否决”。
　　二是完善计划生育兼职委员和领导小组制度，加强部门协作。发挥好计划生育兼职委员和领导小组作用，建立健全重大经济社会政策人口发展影响评估机制，促进相关经济社会政策与计划生育政策的有效衔接。各有关部门认真履行职责，重点解决好政策配套、公共服务保障、执法协调、信息互通等问题，加强对各地工作的指导。深化人口发展战略研究，编制中长期规划。
　　三是强化督导落实，确保政策平稳有序实施。各地各有关部门要按照[《决定》](https://www.pkulaw.com/chl/8564756bae805261bdfb.html)要求，制定实施方案，细化改革任务，明确实施步骤。加强对各项改革措施的跟踪评估，及时发现和解决改革中的苗头性、倾向性问题。各省（区、市）党委政府每年要向中央专题报告本地计划生育工作情况，中央将定期开展督查。国家卫生计生委也将结合对新修订的《[人口与计划生育法](https://www.pkulaw.com/chl/18c6c388fc14eaa9bdfb.html)》落实情况的监督检查工作开展专项督导。

　　**十六、**做好宣传和舆论引导
　　各地要将[《决定》](https://www.pkulaw.com/chl/8564756bae805261bdfb.html)和人口与计划生育法律法规的宣传相结合，广泛开展学习宣传活动，把思想和行动统一到中央的决策部署上来。要深入宣传计划生育工作的伟大成就，大力开展人口基本国情和计划生育基本国策教育，不断增强全社会的国情和国策意识。充分利用广播、电视、报刊、网络等渠道，主动做好政策解读，及时回应社会关切。总结推广改革的好经验、好做法，表彰计划生育先进典型，营造支持改革创新的良好氛围。

　　**十七、**[《决定》](https://www.pkulaw.com/chl/8564756bae805261bdfb.html)核心信息宣传口号
　　1.增强人口国情意识　坚持计划生育基本国策
　　2.实施全面两孩政策　促进人口均衡发展
　　3.实施全面两孩新政　做好基本公共服务
　　4.共创诚信法治计生　共享现代生育文明
　　5.负责任生育　过幸福生活
　　6.生女生男　顺其自然
　　7.关爱女孩就是关注民族的未来
　　8.人口流动　服务相伴
　　9.推进基本公共服务均等化　促进流动人口社会融合
　　10.优生优育　家庭幸福
　　11.关爱妇幼健康　创建幸福家庭
　　12.提高家庭发展能力　促进家庭和谐幸福
　　13.人人健康　家家幸福

© 北大法宝：（[www.pkulaw.com](https://www.pkulaw.com/)) 专业提供法律信息、法学知识和法律软件领域各类解决方案。北大法宝为您提供丰富的参考资料，正式引用法规条文时请与标准文本核对。
欢迎查看所有[产品和服务](https://www.pkulaw.com/helps/2.html)。法宝快讯：[如何快速找到您需要的检索结果？](https://www.pkulaw.com/helps/70.html)[法宝](https://www.pkulaw.com/helps/69.html)V6有何新特色？

扫描二维码阅读原文

**原文链接：**<https://www.pkulaw.com/chl/2d83902c033a0025bdfb.html>
